# From Dead Lithium to Functional Fillers: An in Situ Conversion Strategy for High-Performance All-Solid-State Lithium Metal Batteries

**DOI:** 10.1021/acsami.5c25305

**Published:** 2026-04-07

**Authors:** Guoping Liu, Xinyu Zhang, Maochun Wu

**Affiliations:** Department of Mechanical Engineering, 26680 The Hong Kong Polytechnic University, Kowloon, Hong Kong SAR 999077, China

**Keywords:** PEO solid electrolyte, dead lithium filler, ultrathin thickness, all-solid-state lithium metal batteries

## Abstract

Poly­(ethylene oxide) (PEO) solid electrolytes offer great promise to realize all-solid-state lithium metal batteries with both high energy density and safety. However, it remains challenging to fabricate ultrathin PEO-based solid electrolytes that can operate at practical current densities with a long lifespan. Here, we develop a 19 μm-thick PEO-based solid electrolyte with a porous polyethylene support, which provides mechanical strength and blocks lithium dendrites. By repeatedly plating and stripping lithium at a high current density and low areal capacity, we ingeniously transform otherwise detrimental “dead lithium” into functional fillers within the PEO solid electrolytes. Results show that LiOH, Li_2_CO_3_, Li_2_O, and LiF form on the surface of the “dead lithium”, blocking electronic transport and thus rendering them as effective fillers. These in situ formed fillers simultaneously enhance lithium-ion transport and act as a barrier to suppress dendrite growth, thus facilitating uniform lithium deposition. As a result, this approach enables Li||Li symmetric cells to achieve a critical current density of as high as 1 mA cm^–2^ and operate stably for 400 h at 0.5 mA cm^–2^ and 0.5 mAh cm^–2^ without short-circuits. Importantly, a precycled Li||LiFePO_4_ full cell can retain 90.9% capacity after 600 cycles at 1C charging and 3C discharging.

## Graphical abstract

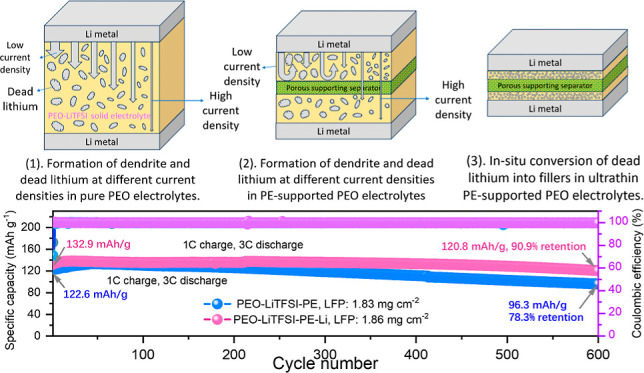

## 1 Introduction

State-of-the-art lithium-ion batteries are approaching their energy density limits, making it difficult to meet the ever-growing demand for high-specific-energy storage devices in emerging fields such as electric aviation, humanoid robots, and long-range electric vehicles.^1,2^ A more pressing concern lies in the inherent safety risks associated with the use of flammable liquid electrolytes, which can trigger thermal runaways, leading to fires or even explosions.^3,4^ All-solid-state lithium metal batteries (ASSLMBs), which use nonflammable solid electrolytes and lithium metal anodes with the highest specific capacity and lowest electrode potential, are considered one of the most promising solutions to meet future energy density and safety requirements for mobile electronic devices and equipment.^5,6^

Solid electrolytes are a key enabling component for ASSLMBs. Among various solid electrolytes, poly­(ethylene oxide) (PEO)-based polymer solid electrolytes stand out as one of the most promising candidates due to their unique advantages, including good interfacial compatibility with lithium metal, high chain flexibility, and excellent processability.^7–9^ However, PEO-based electrolytes suffer from low ionic conductivity and poor mechanical properties, which result in large polarizations and severe lithium dendrite growth.^10,11^ The low ionic conductivity stems primarily from high degree of crystallinity and partly from strong Li^+^-ether oxygen coordination, while the poor mechanical strength results from flexible polymer backbone.^12,13^

To improve the ionic conductivity, various strategies have been proposed, including increasing salt concentration (e.g., polymer in salt), adding plasticizer (e.g., succinonitrile), and incorporating inorganic nanofillers (e.g., Al_2_O_3_, LLZTO).^14–16^ Among these, incorporation of fillers into solid electrolytes has attracted tremendous attention because it can effectively enhance ionic conductivity without sacrificing other properties.^17^ This enhancement arises because the fillers disrupt the ordered arrangement of polymer chains, thus reducing crystallinity, and promote lithium salt dissociation via Lewis acid–base interactions.^3^ Additionally, the presence of fillers helps mitigate lithium dendrite growth by bolstering the mechanical strength of the electrolytes.^18^ Unfortunately, these improvements are often insufficient under rigorous operating conditions. At high areal capacities and/or elevated current densities, lithium dendrites can still penetrate the electrolyte membranes and cause catastrophic short circuits.^17,19^ To suppress dendrite growth, these composite electrolytes are usually fabricated with large thickness, significantly increasing internal resistance and severely diminishing overall battery energy density.

To circumvent this issue, mechanically robust porous scaffolds have been proposed to reinforce PEO-based electrolytes without increasing electrolyte thickness.^20,21^ However, under high current densities, lithium dendrites can still propagate directly through the pores, eventually penetrating electrolyte membranes and causing short circuits.^22,23^ While ceramic fillers can be incorporated in these supported PEO electrolytes to further impede dendrite penetration, it is challenging to ensure the uniform distribution of fillers within the polymer matrix, which may result in uneven current distribution and trigger dendrite growth. These limitations impede the viable application of ultrathin polymer electrolytes under high current density regimes.

In this work, we propose a facile yet highly effective strategy to boost the operating current density of ultrathin polymer electrolytes by combining the advantages of porous support and functional fillers via an in situ conversion approach. Specifically, we develop an ultrathin (19 μm) PEO-based solid electrolyte supported by a porous polyethylene (PE) matrix. By subjecting the cells to high current densities and short plating/stripping periods, we in situ transform otherwise detrimental “dead lithium” into beneficial fillers. Experimental results demonstrate that the dead-lithium-derived fillers are uniformly incorporated into the PEO matrix during cycling, which considerably boosts both the critical current density (CCD) and cycle life of lithium anodes. More importantly, this strategy can be readily translated to full batteries by precycling the battery at a low state of charge. Remarkably, it is demonstrated that a precycled Li||LiFePO_4_ full battery can be stably operated for 600 cycles while maintaining 90.9% of its original capacity, whereas its conventional counterpart suffers from short circuits only after a few cycles.

## 2 Experimental Section

### 2.1 Materials

Poly­(ethylene oxide) (PEO, *M*_w_ = 600,000), anhydrous acetonitrile (ACN, >99.9%), and lithium bis­(trifluoromethane)­sulfonimide (LiTFSI) were provided by Shanghai Aladdin Bio-Chem Technology Co., Ltd. Conductive carbon was obtained from Guangdong Canrd New Energy Technology Co. LiFePO_4_ powder was purchased from Shenzhen Kejing Star Technology Company. The 7 μm PE porous film was purchased from Kuaigoukeyan Company.

### 2.2 Solid-State Electrolyte Preparation

The ultrathin PEO-LiTFSI-PE polymer electrolytes were fabricated through the following steps.

#### 2.2.1 Preparation of PEO-LiTFSI Solution

PEO and LiTFSI with a total mass of 1 g were weighed according to the stoichiometric ratios of O (from PEO) to Li (from LiTFSI) of 8:1, 16:1, 20:1, and 24:1 in an Ar gas glovebox and transferred into a 20 mL glass bottle. ACN was added to adjust the total volume of the solution to 20 mL. The solution was stirred overnight using a magnetic stirrer until it became transparent to obtain the PEO-LiTFSI solution.

#### 2.2.2 Preparation of PEO-LiTFSI-PE Composite Solid Electrolyte Film

The porous PE membrane was cut into a 19 mm-diameter disk, soaked in the PEO-LiTFSI solution for 5 h, then lifted and placed on a PTFE plate. After drying for 48 h, the PEO-LiTFSI-PE solid electrolyte film was obtained by tearing it off the PTFE plate.

#### 2.2.3 In Situ Conversion of Dead Lithium into Filler in PEO-LiTFSI-PE Electrolyte

The obtained PEO-LiTFSI-PE solid electrolyte thin film was sandwiched between two lithium metal disks with a diameter of 12 mm to form a symmetric battery in a coin-cell configuration. The cells were first heated at 60 °C for 10 h in an electric oven. To obtain the in situ generated filler, the cells were cycled at different constant current densities ranging from 0.2 to 0.7 mA cm^–2^ with a short charge and discharge duration of 5 min. The precycling was carried out for 50–150 h to generate dead lithium fillers, which is embedded within the PEO-LiTFSI-PE solid electrolyte. The obtained polymer electrolyte is denoted as PEO-LiTFSI-PE-Li.

### 2.3 Cathode Preparation

In an Ar gas glovebox, 700 mg of lithium iron phosphate (LiFePO_4_) and 100 mg of conductive carbon black were weighed and added to a 20 mL glass bottle. Four milliliters of the PEO-LiTFSI solution (corresponding to a solid content of 200 mg of PEO-LiTFSI) was added into the glass bottle and magnetically stirred overnight to obtain the cathode slurry. The slurry was then uniformly cast on a carbon-coated aluminum foil and dried for 24 h to obtain the LiFePO_4_ cathode sheet. The cathode sheet was cut into 10 mm-diameter disks before use.

### 2.4 Li||LiFePO_4_ Full Cell Assembly

The coin-type Li||LiFePO_4_ batteries were assembled by stacking a lithium metal anode, the PEO-LiTFSI-PE electrolyte, and a LiFePO_4_ cathode. To generate the in situ formed filler in the PEO-LiTFSI-PE electrolyte, the battery was first heated at 60 °C for 24 h and then charged at 0.5C for 1 h. The battery was then charged and discharged at a current density of 0.5 mA cm^–2^ with a period of 5 min for 300 cycles. Afterward, the battery was fully charged at 0.5C to 4.2 V and maintained at a constant voltage until the current was less than 0.05C. At this point, the in situ conversion of dead lithium into filler was completed in full batteries, and the battery is denoted as Li||PEO-LiTFSI-PE-Li||LiFePO_4_.

### 2.5 Electrochemical Measurements

Electrochemical impedance spectroscopy (EIS) and linear sweep voltammetry (LSV) tests were conducted on an electrochemical workstation (Biologic, SP-300). For EIS tests, the frequency range was set from 7 MHz to 1 mHz and the amplitude was 10 mV. LSV was performed by sweeping the potential (vs Li^+^/Li) from 3.0 to 6.5 V at a scan rate of 1 mV s^–1^. Constant current tests were performed on a Neware battery testing system. For critical current density (CCD test, Li||Li symmetric cells were cycled by increasing the current density from 0.05 to 1.0 mA cm^–2^ at an increment of 0.05 mA cm^–2^. The cells were cycled at each current density 5 times, and each cycle lasted 30 min for charging and discharging. For full battery tests, the cutoff voltages were set at 2.5–4.2 V. For rate performance tests, the batteries were charged at 0.5C and discharged at 0.5–3C. For the cycling performance test, the batteries were first charged at constant rates of 0.5–1C to a cutoff voltage of 4.2 V, held at this constant voltage until the current was less than 0.05C, and discharged at different rates.

### 2.6 Materials Characterizations

The diffraction patterns of the PE and PEO-based solid electrolyte samples were collected on a Bruker D8 Advance powder diffractometer equipped with Cu Kα radiation (λ = 1.541 Å). Field-emission scanning electron microscopy (SEM, Hitachi SU8220) was used to analyze the morphology of the PE and PEO-based solid electrolyte samples. X-ray photoelectron spectroscopy (XPS, Thermo Fisher Escalab 250Xi) analysis was conducted to identify the bonding information on the PEO-based solid electrolyte. Tensile tests were conducted using a QT6203S universal testing machine (Qian Tong Instrument Equipment Co., Ltd., Jiangsu, China).

## 3 Results and Discussion

Figure 1a illustrates the formation of lithium dendrites and “dead lithium” in different PEO-based electrolytes under various current densities. In general, lithium dendrites are coarser and grow more slowly at lower current densities, making it difficult to penetrate solid electrolytes. Conversely, elevated current densities accelerate lithium dendrite growth and reduce their diameter, facilitating rapid penetration through the PEO solid electrolytes and resulting in premature short circuits (Figure S1). Consequently, conventional PEO-LiTFSI solid electrolytes require substantial thickness (>100 μm) to suppress dendrite penetration, which significantly sacrifices the battery energy density. By adding a porous PE layer as mechanical support, the electrolyte thickness can be significantly reduced while maintaining resistance to lithium dendrite penetration at low current densities. However, severe dendrite proliferation at high current densities still results in membrane penetration and cell failure (Figure S2). It is widely recognized that during stripping, dendritic lithium electrodeposits often undergo mechanical fracture at their roots, electrically disconnecting from the electrodes to form isolated “dead lithium”. Given its high reactivity, the resulting dead lithium reacts with the surrounding electrolytes to form an electrically passivated layer. We propose that, by judiciously tailoring the cycling conditions, it is possible to tune the size of dead lithium and transform them into functional inorganic fillers even with a small electrolyte thickness, which can effectively enhance the ionic conductivity and suppress dendrite formation.

**Figure 1:**
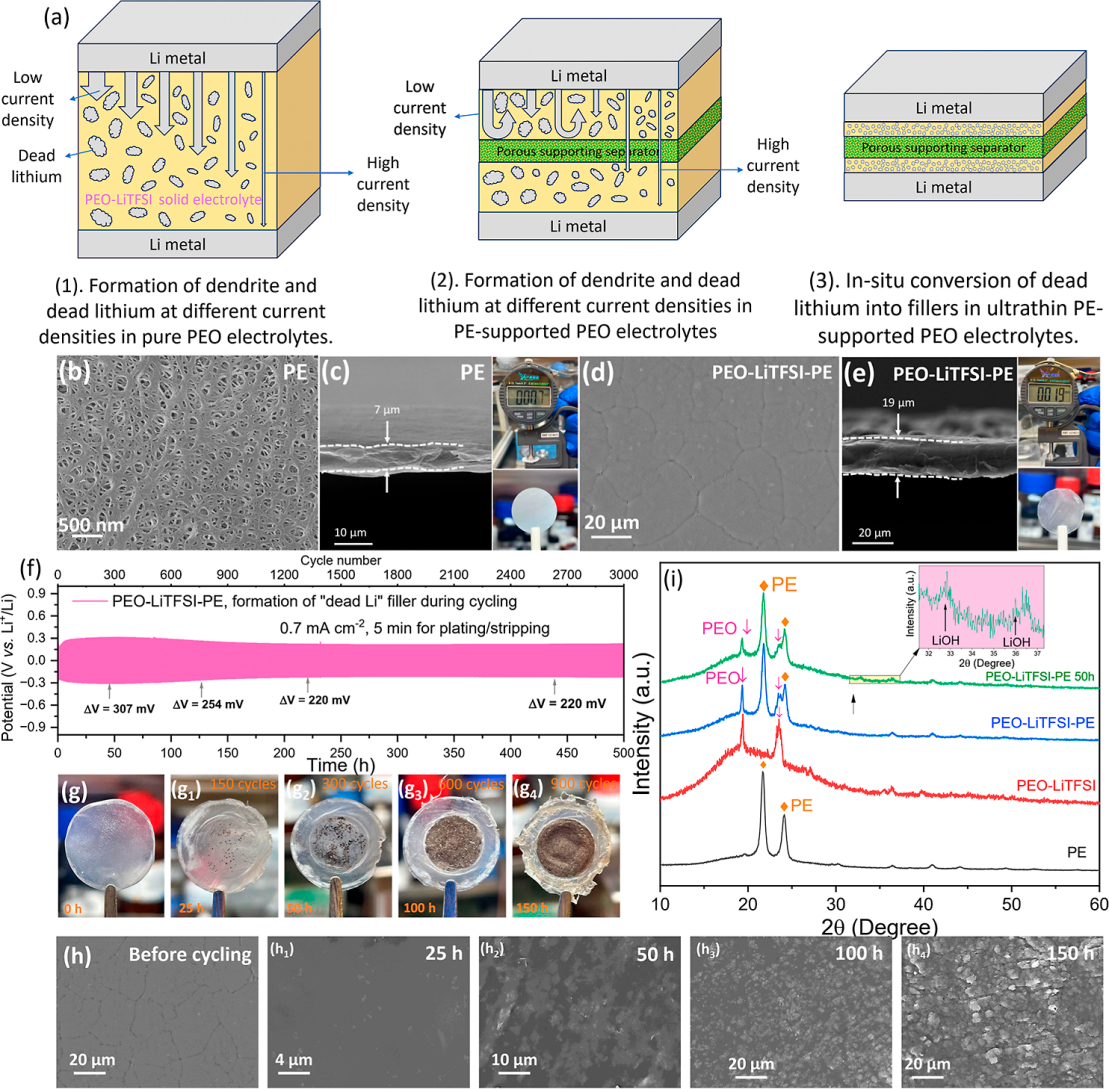
(a) Schematic illustration of lithium dendrite growth and dead lithium formation in different PEO-based electrolytes. (b) Surface-section SEM image and (c) cross-section SEM image and digital photos of PE separator. (d) Surface-section SEM image and (e) cross-section SEM image and digital photos of PEO-LiTFSI-PE. (f) Voltage profiles of a Li||Li cell cycled at 0.7 mA cm^–2^ with 5-min charge/discharge periods. (g) Digital photos and (h) SEM images of PEO-LiTFSI-PE and PEO-LiTFSI-PE-Li electrolytes during different cycle periods. (i) XRD patterns of the PE, PEO-LiTFSI, PEO-LiTFSI-PE, and PEO-LiTFSI-PE-Li electrolytes cycled after 50 h.

To verify this hypothesis, we first fabricated ultrathin PEO solid electrolytes reinforced with a PE scaffold. As shown in Figure 1b, the surface morphology of the pristine PE support shows an interconnected fibrous network with abundant nanopores, which can enhance the mechanical strength of polymer electrolytes. Moreover, the pore size of the PE support ranges in tens of nanometers, effectively blocking the penetration of large-sized lithium dendrites.SEM image and vernier caliper confirm that the free-standing PE support has a thickness of only 7 μm (Figure 1c). After filling with PEO-LiTFSI electrolytes, the surface becomes dense and smooth (Figure 1d), indicating successful infiltration of polymer electrolyte into the nanoporous network. Figure 1e confirms that the resulting PE-supported PEO-LiTFSI (PEO-LiTFSI-PE) electrolyte features a total thickness of merely 19 μm while retaining free-standing characteristics, which are important for scale-up manufacturing and practical battery assembly.

We then assembled a symmetric Li||Li cell using the LiTFSI–PEO-PE electrolyte and cycled it at a high current density of 0.7 mA cm^–2^ with 5-min charging and 5-min discharging to in situ generate dead lithium fillers (the resulting electrolyte is denoted as PEO-LiTFSI-PE-Li).^22^ To reduce the crystallinity of PEO and better optimize the performance of the solid-state electrolyte, the tests were conducted at 60 °C. As shown in Figure 1f, during the initial cycling stage, the cell exhibits relatively large polarizations and the voltage difference between charge and discharge (Δ*V*) continues to rise to 307 mV over the first 45 h. Subsequently, it gradually drops to 254 mV at 125 h and stabilizes at 220 mV after 220 h. This distinct voltage stabilization is probably attributed to the enhanced ionic conductivity resulting from the in situ formation of dead lithium fillers. To visualize the evolution of dead lithium fillers during cycling, we disassembled the cells at different cycling intervals to examine the solid electrolytes. Optical images (Figure 1g) reveal that the pristine PEO-LiTFSI-PE membrane is initially transparent. After 25 h of cycling, black deposits emerge within the matrix, indicating the formation of dead lithium.^24^ As cycling progresses to 50 and 100 h, the density of these black fillers significantly increases. Ultimately, after 150 h, the dead lithium fillers achieve a completely uniform, dense distribution throughout the entire PEO-LiTFSI-PE solid electrolyte.

SEM was employed to observe the microscopic morphology of the in situ generated fillers. As shown in Figure 1h, the surface of the pristine PEO-LiTFSI-PE solid electrolyte has distinct regional boundaries. After cycling for 25 h, the electrolyte surface becomes smoother and some nanoparticles (i.e., dead lithium) appear. The disappearance of the regional boundary indicates that the formation of dead lithium fillers contributes to homogenizing the surface of the solid-state electrolyte. With cycling prolonged to 100 h, the density of dead lithium fillers gradually increases, forming a compact, uniform morphology. By 150 h, micrometer-sized lithium particles emerge on the electrolyte surface, which likely originate from active lithium near the lithium electrode interface. These observations indicate that 100 h of precycling is optimal for generating uniformly distributed dead lithium fillers within the polymer electrolytes. To determine the phase composition of the dead lithium fillers, X-ray crystallography (XRD) was performed. As shown in Figure 1i, the pristine PEO-LiTFSI-PE solid electrolyte exhibits characteristic diffraction peaks of PE (21.7° and 24.1°) and PEO-LiTFSI (19.4° and 23.5°). After 50 h of cycling, the intensity of PEO-LiTFSI peaks decreases, indicating that the dead lithium fillers reduce the crystallinity of PEO-LiTFSI. Furthermore, two new peaks emerge at 32.7° and 36.0° after 50 h of cycling, corresponding to LiOH, which is a common component in solid-electrolyte interphase (SEI). Notably, no diffraction peaks of metallic lithium are detected. This can be attributed to its nanoscale size and high specific surface area, which causes it to readily react with the PEO electrolytes, forming passivating SEI layers. Figure S3 compares the mechanical strength of PEO-LiTFSI and PEO-LiTFSI-PE-Li via tensile tests. The yield strength of the pristine PEO-LITFSI solid-state electrolyte is only 5.7 MPa. After the addition of PE, the yield strength of PEO-LITFSI increases to 164.2 MPa, indicating that the incorporation of PE significantly enhances the tensile strength of the solid-state electrolyte.

XPS was employed to further characterize the chemical composition of the dead lithium fillers. As shown in Figure S4, the pristine PEO-LiTFSI-PE solid-state electrolyte exhibits characteristic signals for C, O, F, N, S, and Li. After 50 cycles of cycling to form dead lithium fillers, we performed depth-profiling XPS on the resulting PEO-LiTFSI-PE-Li. As shown in Figure 2, the high-resolution C 1s spectrum reveals a distinct peak at 288.2 eV corresponding to the C=O double bond, indicating the formation of Li_2_CO_3_ on the filler surface via reaction between the dead lithium and the electrolyte. Furthermore, the high-resolution F 1s, N 1s, and O 1s spectra display signals of Li–F, Li–N, and Li–O at 685.1, 398.6, and 140.3 eV, respectively, corresponding to the LiF, Li_3_N, and Li_2_O components in the SEI. Quantitative elemental analysis (Table S1) shows that the atomic concentrations of F, N, and O are 10.24%, 1.11%, and 19.58%, respectively, indicating that the content of Li_3_N in the SEI on the dead lithium surface is very low. Notably, after etching, the intensity of the Li–F signal significantly increases, suggesting an enrichment of LiF content in the deeper regions of the SEI, whereas the Li_2_O and Li_2_CO_3_ contents remain largely unchanged.

**Figure 2:**
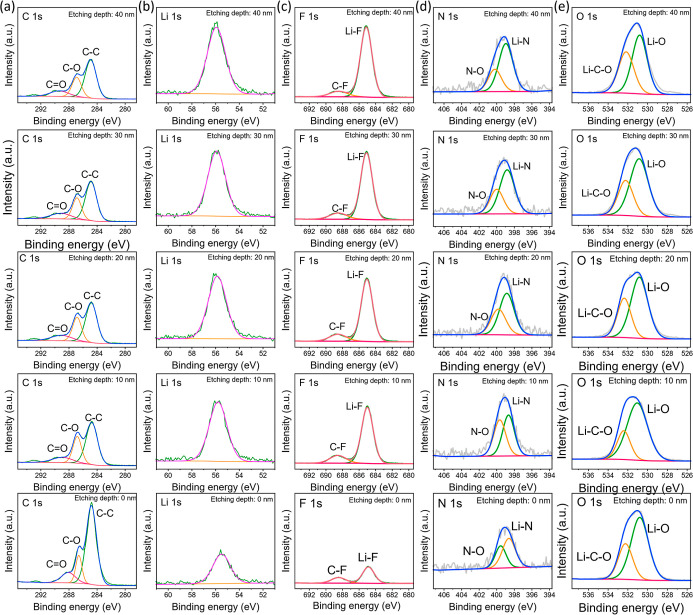
High-resolution XPS spectra of (a) C 1s, (b) Li 1s, (c) F 1s, (d) N 1s, and (e) O 1s of PEO-LiTFSI-PE-Li electrolyte at different etching depths.

Based on these results, the evolved solid electrolyte with functional fillers after cycling is schematically illustrated in Figure 3a. Specifically, the centrally positioned PE scaffold serves as a robust structural support, while the solid electrolytes on both sides become progressively enriched with in situ generated dead lithium fillers during repeated cycling. The incorporation of these fillers reduces the crystallinity of PEO-LiTFSI electrolyte, thereby increasing the ionic conductivity. Moreover, the SEI components (LiOH, Li_2_CO_3_, Li_2_O, and LiF) encapsulating these fillers undergo Lewis acid–base interactions with lithium salts, further facilitating rapid lithium-ion transport. Notably, the dead lithium fillers formed during the initial cycling stages are smaller and predominantly distributed near the PE support (Figure 3b,c), whereas larger dead lithium particles emerge near the lithium electrode interface during later stages (Figure 3d). This gradient distribution is highly advantageous, as it provides ample space for subsequent lithium deposition and effectively mitigates dendrite-induced short circuits. To verify the improvement of electrochemical performance after the formation of dead lithium fillers, we evaluated the rate performance of symmetric cells following a 132-h precycling step to generate the fillers. As shown in Figure 3e, the cell delivers a high CCD of 0.9 mA cm^–2^. When the current density is further increased to 0.95 mA cm^–2^, the cell suffers from significant polarizations (0.71 V). To further increase the CCD, we optimized the precycling protocol by first repeatedly 5-min plating and 5-min striping of lithium at 0.7 mA cm^–2^ for over 63 h, followed by cycling the cell at a longer plating/stripping period of 30 min for 50 h to generate larger dead lithium particles. This tailored protocol successfully boosts the CCD of symmetric cells to 1.0 mA cm^–2^ (Figure S5).

**Figure 3:**
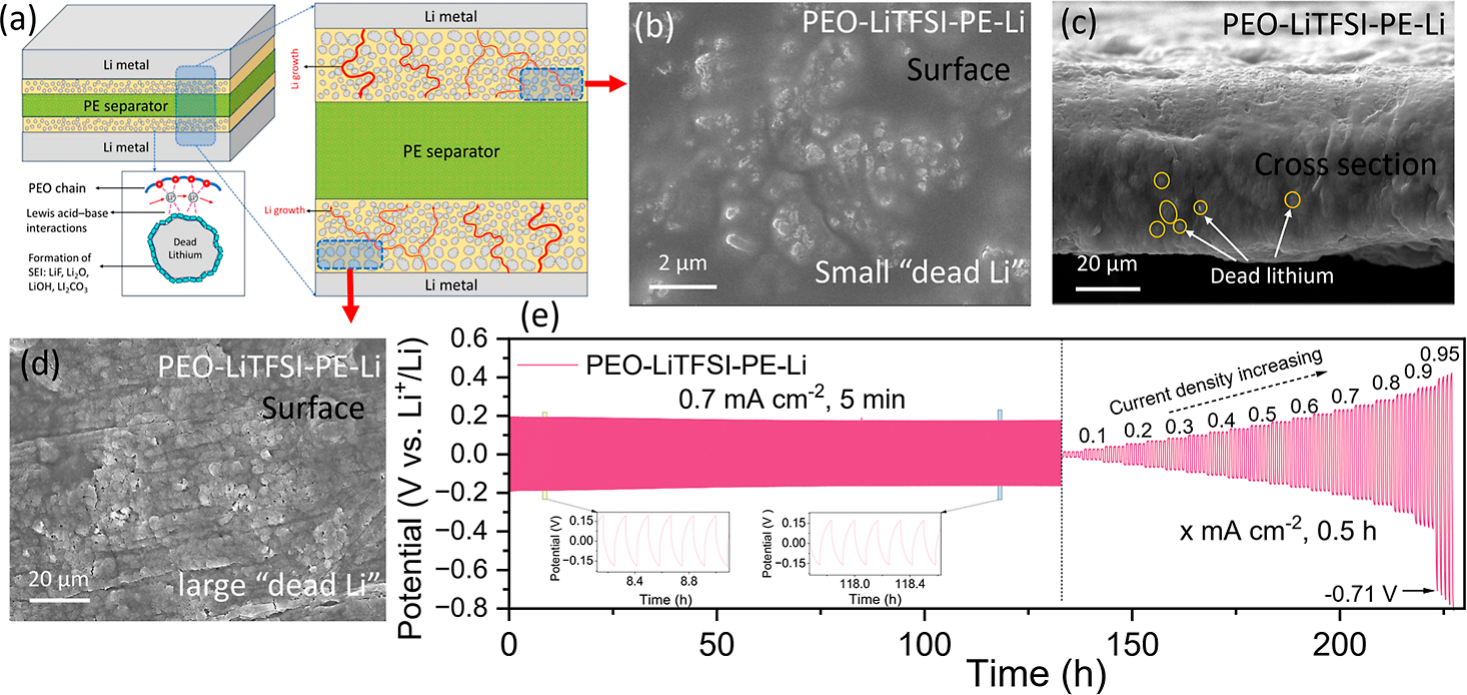
(a) Schematic diagram of the distribution of dead lithium fillers in PEO-LiTFSI-PE-Li and its interaction with lithium ions. (b) Small dead lithium fillers of the PEO-LiTFSI-PE-Li after cycling for 50 h. (c) Cross-section SEM image of the PEO-LiTFSI-PE-Li after cycling for 100 h. (d) Large dead lithium filler of the PEO-LiTFSI-PE-Li after cycling for 150 h. (e) Critical current density test after formation of dead lithium fillers of PEO-LiTFSI-PE-Li.

For better comparison, the CCD test result is presented in Figure 4a and compared with those of pristine PEO-LiTFSI-PE and PEO-LiTFSI electrolytes (Figure 4b,c). As displayed in Figure 4c, the cell with conventional PEO-LiTFSI electrolyte can operate stably at current densities less than 0.2 mA cm^–2^. Exceeding 0.25 mA cm^–2^ triggers micro-short circuits, signaling onset of lithium dendrite penetration. Continued cycling inevitably leads to hard short circuits and complete cell failure. These results indicate that unsupported PEO electrolytes are highly susceptible to unmitigated dendrite proliferation, consistent with previous results in open literature. Figure 4b shows that the integration of a PE support effectively elevates the CCD to 0.35 mA cm^–2^ without micro-short circuits. Although further increasing the current increases to 0.4–0.5 mA cm^–2^ induces micro-short circuits, the cell avoids a catastrophic hard short. This highlights the ability of PE scaffold to maintain macroscopic physical separation between the two electrodes and preserves overall structural integrity. Nevertheless, at high current densities, severe localized dendrite growth still propagates through the porous PE structure, causing micro-short circuits. In stark contrast, the PEO electrolyte with in situ formed dead lithium fillers enables the cell to operate at a current density as high as 1.0 mA cm^–2^ without any short circuits (Figure 4a), confirming the effectiveness of our strategy in boosting the operating current density of ultrathin PEO-based solid electrolytes. Figure 4d summarizes the CCD of Li||Li cells with PEO-LiTFSI, PEO-LiTFSI-PE, and PEO-LiTFSI-PE-Li, which are 0.2, 0.35, and 1.0 mA cm^–2^, respectively. Notably, the CCD of PEO-LiTFSI-PE-Li surpasses most reported solid electrolytes (Figure 4e). It should be noted that this exceptional performance is achieved with an ultrathin electrolyte layer (<20 μm), whereas previously reported solid electrolytes are typically 76–300 μm thick, further demonstrating the superiority of the proposed strategy in tackling the challenges of PEO electrolytes. In addition, we investigated the effect of lithium salt concentrations on limiting current density. As shown in Figure S6, the CCD values for PEO_8_–LiTFSI-PE-Li, PEO_16_–LiTFSI-PE-Li, PEO_20_-LiTFSI-PE-Li, and PEO_24_–LiTFSI-PE-Li are 0.3, 0.8, 1.0, and 0.65 mA cm^–2^, respectively, identifying PEO_20_-LiTFSI-PE-Li as the optimal formulation. Furthermore, to examine the applicability at near room temperature, we tested the half-cell performance of PEO_20_-LiTFSI-PE-Li at 30 °C. As displayed in Figure S7, as the current density was increased from 0.01 to 0.1 mA cm^–2^, the overpotential rose from 0.11 to 1.23 V without short-circuiting. This result suggests that PEO_20_-LiTFSI-PE-Li maintains a certain level of practical viability even at ambient temperature.

**Figure 4:**
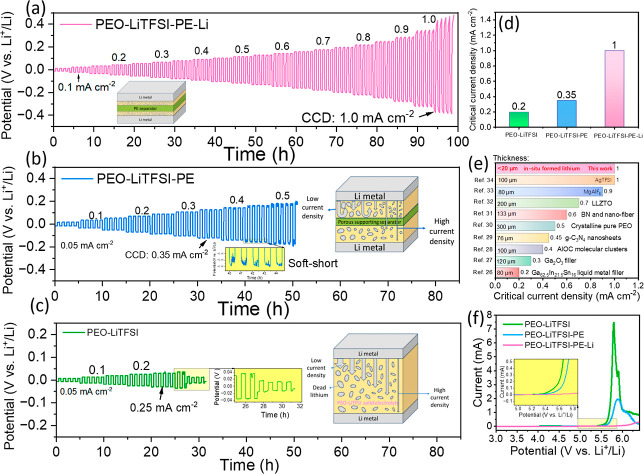
Potential-time profiles of Li||Li cells using (a) PEO-LiTFSI-PE-Li, (b) PEO-LiTFSI-PE, and (c) PEO-LiTFSI electrolytes with increasing current densities. (d) CCD of the cells with PEO-LiTFSI, PEO-LiTFSI-PE, and PEO-LiTFSI-PE-Li electrolytes. (e) Comparison of CCD and thickness of the PEO-LiTFSI-PE-Li withthe reported polymer-based electrolytes.^26–34^ (f) LSV curves of the PEO-LiTFSI, PEO-LiTFSI-PE, and PEO-LiTFSI-PE-Li electrolytes.

Given that inorganic fillers are well-known to broaden the electrochemical window of polymer electrolytes, we also performed LSV to evaluate the stability of the different PEO-based electrolytes. Figure 4f shows the LSV profiles of PEO-LiTFSI, PEO-LiTFSI-PE, and PEO-LiTFSI-PE-Li solid electrolytes over a potential range of 3.0–6.5 V. While all three electrolytes exhibit negligible oxidation below 5 V, their high-voltage behaviors differ significantly. For conventional PEO-LiTFSI electrolyte, a pronounced anodic current emerges at 5.3 V, escalating to a peak of 7.5 mA at 5.8 V. The PEO-LiTFSI-PE electrolyte shows a slightly delayed oxidation onset at 5.4 V and a suppressed peak current of 1.9 mA at 5.9 V. This may be because the inactive PE scaffold replaces a portion of oxidation-prone PEO, thereby mitigating the overall parasitic current. In striking contrast, the PEO-LiTFSI-PE-Li electrolyte exhibits exceptional anodic stability, with oxidation onset delayed to 5.8 V and a remarkably low current of only 0.3 mA even at 6.5 V. These results confirm that in situ conversion of dead lithium into fillers can effectively enhance the oxidation stability of the PEO-based electrolytes, which is crucial for practical applications. The substantial enhancement of oxidation potential can be attributed to a dual mechanism effect by the in situ formed dead lithium fillers. First, the dead lithium likely scavenges the terminal hydroxyl groups (OH) of PEO chains. Because these −OH groups are widely considered the root cause of PEO decomposition at high potentials, their elimination fortifies the polymer backbone against oxidation.^25^ Second, the in situ formation of highly stable LiF on the surface of dead lithium further enhances the oxidation resistance of the electrolytes.

Figure 5a shows the Tafel curves of Li||Li cells with PEO-LiTFSI, PEO-LiTFSI-PE, and PEO-LiTFSI-PE-Li electrolytes within a potential range of −0.15 to 0.15 V. The pristine PEO-LiTFSI electrolyte exhibits the highest exchange current density of 0.64 mA cm^–2^, while the addition of PE support reduces this value to 0.10 mA cm^–2^. This is because the nonconductive porous PE support increases the tortuosity and lengthens the lithium-ion transport path. However, after in situ generation of dead lithium fillers, the exchange current density increases to 0.24 mA cm^–2^, indicating that the functional fillers facilitate ionic conduction and improve interfacial kinetics. To gain a deeper understanding of lithium deposition behavior, lithium plating was conducted at a current density of 0.1 mA cm^–2^ using different electrolytes. As shown in Figure 5b, the plating overpotential increases in the sequence of PEO-LiTFSI < PEO-LiTFSI-PE-Li < PEO-LiTFSI-PE, which is consistent with the results acquired from Tafel curves. EIS tests were subsequently performed to deconvolute the origin of resistances. The corresponding Nyquist plots (Figure 5c) reveal that conventional PEO-LiTFSI shows the lowest overall impedance (55.5 Ω), comprising an ohmic impedance of 6.4 Ω and an interfacial impedance of 28.5 Ω. As expected, the incorporation of the PE matrix inherently leads to a substantial increase in impedance due to the restricted and highly tortuous lithium-ion transport pathways within the porous framework. Notably, the PEO-LiTFSI-PE-Li yields a markedly lower total impedance (260.2 Ω) compared to PEO-LiTFSI-PE, featuring an ohmic impedance of 22.0 Ω and an interfacial impedance of 59.5 Ω. This notable impedance reduction is attributed to the generation of dead lithium fillers, which reduces PEO crystallization and promotes lithium salt dissociation, thereby enhancing the ionic conductivity. These results are consistent with the previous test results.

**Figure 5:**
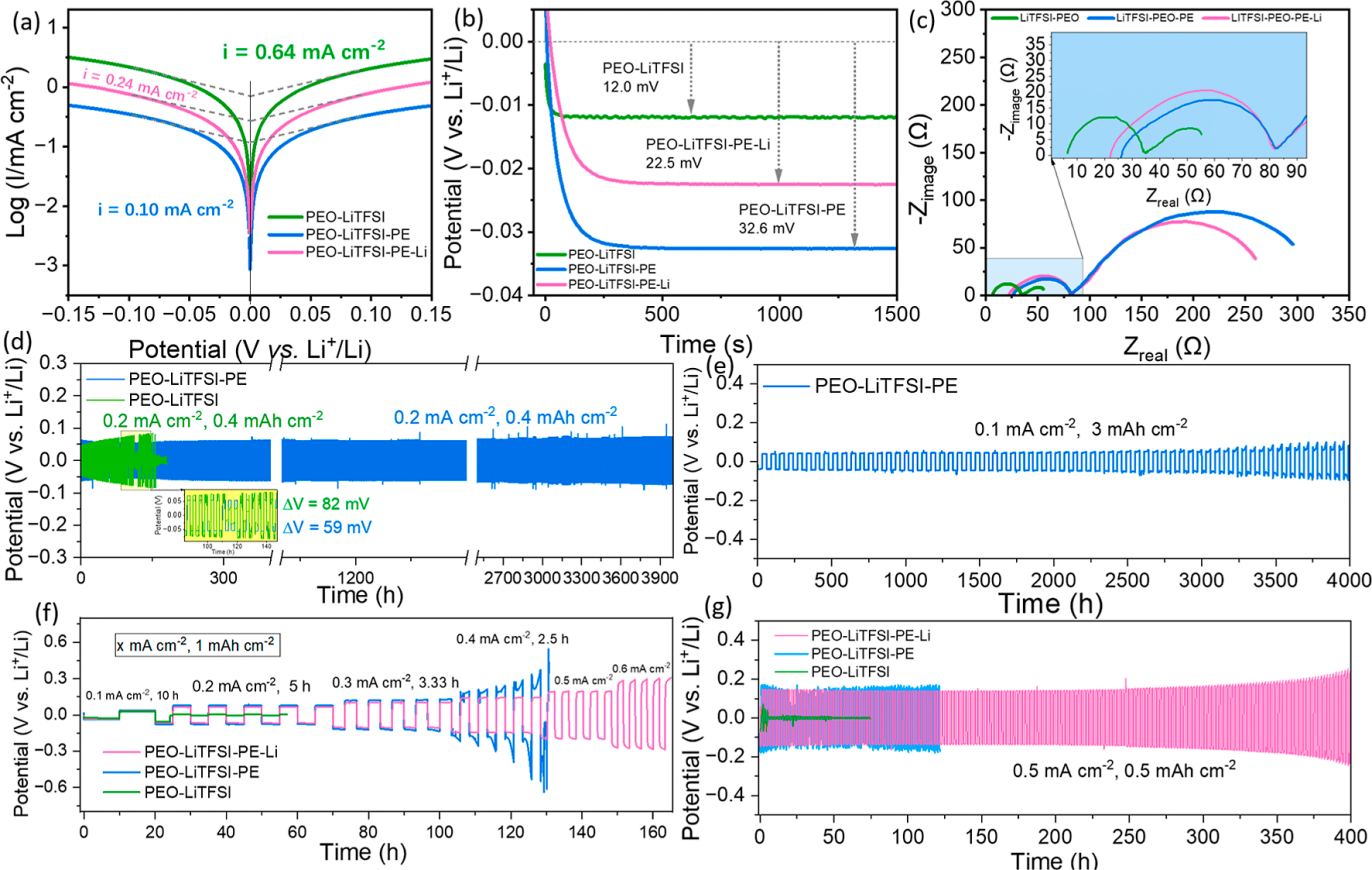
(a) Tafel plots and exchange current densities of the PEO-LiTFSI, PEO-LiTFSI-PE, and PEO-LiTFSI-PE-Li electrolytes. (b) Potential-time curves of cells using PEO-LiTFSI, PEO-LiTFSI-PE, and PEO-LiTFSI-PE-Li electrolytes during plating at 0.1 mA cm^–2^. (c) Nyquist plots of PEO-LiTFSI, PEO-LiTFSI-PE, and PEO-LiTFSI-PE-Li. (d) Galvanostatic cycling curves of PEO-LiTFSI, PEO-LiTFSI-PE symmetric cells at 0.2 mA cm^–2^ and 0.4 mAh cm^–2^. (e) Galvanostatic cycling curves of PEO-LiTFSI-PE symmetric cells at 0.1 mA cm^–2^ and 3 mAh cm^–2^. (f) Rate performance of lithium symmetric cells using PEO-LiTFSI, PEO-LiTFSI-PE, and PEO-LiTFSI-PE-Li with a fixed areal capacity of 1 mAh cm^–2^. (g) Galvanostatic charge–discharge curves of Li||Li cells with PEO-LiTFSI, PEO-LiTFSI-PE, and PEO-LiTFSI-PE-Li electrolytes at 0.5 mA cm^–2^ and 0.5 mAh cm^–2^.

We further evaluated the long-term stability of Li||Li cells with various electrolytes by performing galvanostatic cycling at different current densities and areal capacities. Figure 5d shows the voltage profiles at 0.2 mA cm^–2^ and 0.4 mAh cm^–2^. The cell using PEO-LiTFSI electrolyte exhibits erratic voltage fluctuations indicative of micro-short circuits after only 50 h, accompanied by a progressive increase in overpotential, culminating in a hard short circuit after 160 h. In contrast, the use of PEO-LiTFSI-PE electrolyte enables the lithium symmetric cells to cycle stably for 4000 h with a relatively low and stable Δ*V* of 59 mV. This indicates that the addition of PE can effectively suppress dendrite growth at lower current densities. Notably, even at a high areal capacity of 3 mAh cm^–2^, the cell with PEO-LiTFSI-PE electrolyte can stably operate for 4000 h (Figure 5e). To further probe the rate capability, symmetric cells were subjected to stepped current density at a fixed areal capacity of 1 mAh cm^–2^ (Figure 5f). The conventional PEO-LiTFSI cell failed quickly at 0.2 mA cm^–2^ due to severe dendrite penetration. While the PE-supported electrolyte sustained stable cycling at 0.3 mA cm^–2^ without short circuits, it succumbs to micro-short circuits when the current density is elevated to 0.4 mA cm^–2^, indicating that the use of porous PE support cannot halt dendrite penetration at higher current densities. In stark contrast, the PEO-LiTFSI-PE-Li electrolyte enables the cell to cycle stably even at a current density of 0.6 mA cm^–2^, indicating that the introduction of dead lithium fillers considerably boosts the rate performance of the battery under large areal capacity. The long-term cycling durability was further evaluated under more stringent conditions (0.5 mA cm^–2^ and 0.5 mAh cm^–2^). As displayed in Figure 5g, a symmetric cell with PEO-LiTFSI can hardly operate under these conditions due to rampant growth of lithium dendrites at higher current densities. The PEO-LiTFSI-PE cell also exhibits micro-short circuits during the initial deposition process of electrolyte and persists with continuous cycling. This may be due to the aggressive dendrite propagation through the interconnected pores of the PE framework. Remarkably, the cell with PEO-LiTFSI-PE-Li electrolyte sustains over 400 h of cycling without short circuits, further confirming the effectiveness of in situ formed dead lithium fillers in elevating the long-term cycling stability of ultrathin solid polymer electrolytes.

To verify the practicality of the proposed strategy in full batteries, Li||LiFePO_4_ full batteries were assembled and tested. SEM images of LiFePO_4_ cathode (Figure 6a,b) reveal a flat and uniform topography, comprising LiFePO_4_ active material particles smaller than 2 μm. Cross-section SEM analysis (Figure 6c–f) demonstrates intimate interfacial contact between the solid electrolyte and the LiFePO_4_ positive electrode, with no observable voids or delamination. Corresponding elemental mapping at the interface (Figure 6e,f) further delineates this architecture, confirming a solid electrolyte layer with a thickness of 14 μm, which is due to the infiltration of partial solid electrolyte into the positive electrode during the heating and assembly process. To quantify the interfacial resistances, EIS of both Li||Li half cells and Li||LiFePO_4_ full cells were tested. As shown in Figure S8a, the overall interfacial impedance is 59.3 Ω in the Li||Li half-cell and 49.7 Ω in the full cell. By deconvoluting these values, the interfacial resistance between the solid electrolyte and the positive electrode is calculated to be as low as 5.5 Ω cm^2^ (Figure S8b).

**Figure 6:**
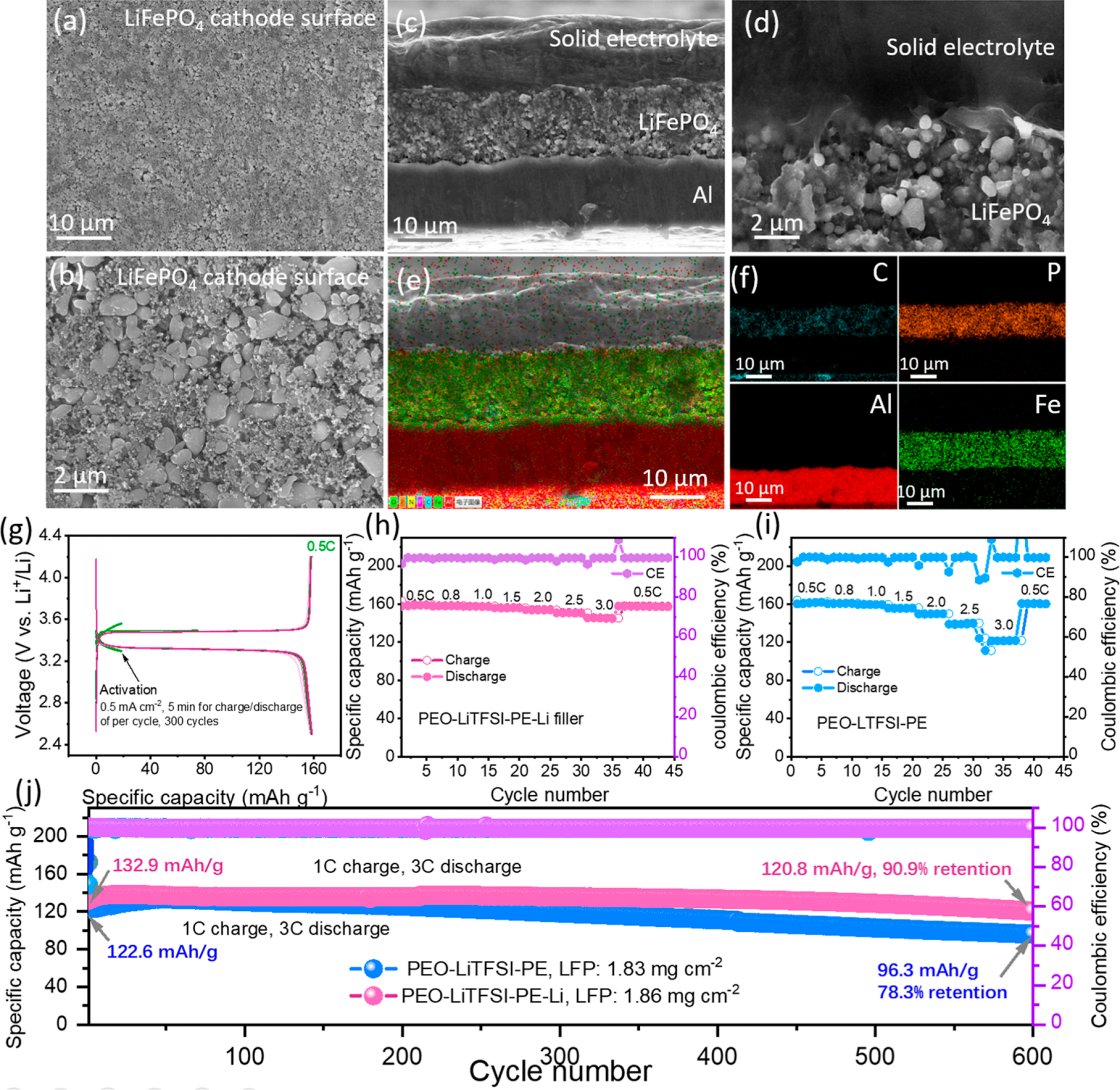
(a,b) SEM images of LiFePO_4_ cathode. (c,d) Cross-section SEM images of integrated PEO-LiTFSI-PE||LiFePO_4_ cathode. (e,f) Cross-section mapping results of integrated PEO-LiTFSI-PE||LiFePO_4_ cathode. (g) Charge–discharge curves of precycles for in situ formation of dead lithium fillers and normal operation of Li||LiFePO_4_ full cell. Rate performance of (h) Li||PEO-LiTFSI-PE-Li||LiFePO_4_ and (i) Li||PEO-LiTFSI-PE||LiFePO_4_ full cells. (j) Cycling performance of Li||PEO-LiTFSI-PE-Li||LiFePO_4_ and Li||PEO-LiTFSI-PE||LiFePO_4_ full cells.

To in situ form dead lithium fillers within the full-cell configuration, the battery was precycled at high current density (0.5 mA cm^–2^) with short-term charge–discharge cycles (10 min per cycle) for 50 h (Figures 6g and S9). Following the precycles, the battery was charged and discharged at a rate of 0.5C. As shown in Figure 6g, the battery delivers a specific charge capacity of 158.9 mAh g^–1^ and a specific discharge capacity of 158.8 mAh g^–1^, yielding a Coulombic efficiency of 99.94%. Figure 6h, i presents the rate performance of Li||LiFePO_4_ full cells using PEO-LiTFSI-PE and PEO-LiTFSI-PE-Li solid electrolytes. During the tests, the charge rate was fixed at 0.5C, while the discharge rate varied from 0.5 to 3C. While capacity inherently decays at higher rates for both cells, the battery with PEO-LiTFSI-PE-Li electrolyte still delivers a specific capacity of 145.9 mAh g^–1^ at 3C, significantly higher than 121.5 mAh g^–1^ for its counterpart with PEO-LiTFSI-PE. Figure S10 shows the schematic diagram of the full battery structure and corresponding charge and discharge curves. With the increase of discharge rate, the battery with PEO-LiTFSI-PE-Li exhibits lower polarizations and higher specific capacity than that with PEO-LiTFSI-PE. Figure 6j shows the long-term cycling performance of full batteries with PEO-LiTFSI-PE and PEO-LiTFSI-PE-Li, with a charge rate of 1C and a discharge rate of 3C. The initial discharge capacity of the battery with PEO-LiTFSI-PE-Li electrolyte is 132.9 mAh g^–1^, which is higher than that with PEO-LiTFSI-PE (122.6 mAh g^–1^). After 600 cycles, the battery with PEO-LiTFSI-PE-Li can still maintain a specific capacity of 120.8 mAh g^–1^, corresponding to a capacity retention rate of 90.9%, in stark contrast to 96.3 mAh g^–1^ (78.3% capacity retention rate) of the battery with PEO-LiTFSI-PE. Postcycling EIS test on the Li||PEO-LiTFSI-PE-Li||LiFePO4 full cell (Figure S11) reveals that after 100 cycles, the ohmic and interfacial impedance drops to 13.3 Ω and 44.2 Ω, respectively, both lower than their precycling values. Furthermore, the cycling performance of the Li||PEO-LiTFSI-PE-Li||LiFePO_4_ full cell was tested at 30 °C. As shown in Figure S12, at a charge–discharge rate of 0.1C, the full cell delivers a reversible capacity of 85.8 mAh g^–1^, which gradually increases to 132.2 mAh g^–1^ by the 35th cycle and then decreases to 95.6 mAh g^–1^ by the 140th cycle. This indicates that the Li||PEO-LiTFSI-PE-Li||LiFePO_4_ full cell is also capable of operating at near room temperature. However, the performance is much inferior compared to elevated temperature (i.e., 60 °C), which is a long-standing challenge of PEO-based electrolyte. To demonstrate the practicability of the developed strategy, cell performance was evaluated with a high active mass loading of 7.8 mg cm^–2^. Impressively, the battery with PEO-LiTFSI-PE-Li is still able to deliver an initial specific capacity of 159.2 mAh g^–1^ at 0.1C and maintain 157.0 mAh g^–1^ after 60 cycles without short circuits at 60 °C (Figure S13). Conversely, the control cell with PEO-LiTFSI solid electrolyte suffers from a short circuit during the first cycle of charging (Figure S14). In the subsequent charging process, short circuits consistently exist, confirming that PEO-LiTFSI electrolyte is not compatible with high-mass-loading cathodes. These results demonstrate the great potential of in situ generation of dead lithium fillers for practical applications.

## 4 Conclusion

In summary, we have developed an ultrathin PEO-based solid electrolyte and established a facile strategy to in situ convert dead lithium into functional fillers to boost the performance of solid-state lithium batteries. Material characterizations confirm that a SEI consisting of LiOH, Li_2_CO_3_, Li_2_O, and LiF is formed on the dead lithium, blocking the electronic transport and thus rendering dead lithium as inorganic fillers. It is demonstrated that the fillers effectively enhance the ionic conductivity due to the Lewis acid–based interaction and suppress dendrite growth. Consequently, the resulting PEO-LiTFSI-PE-Li electrolytes enable a lithium symmetric cell to achieve a high critical current density of 1 mA cm^–2^. Moreover, the proposed method can be readily implemented in full batteries. The newly developed Li||PEO-LiTFSI-PE-Li||LiFePO_4_ full battery achieves a capacity retention rate of 90.9% after 600 cycles under 1C charging and 3C discharging conditions. More remarkably, the battery can still operate stably under a high active mass loading of 7.8 mg cm^–2^. This method provides a new avenue for the development of high-performance all-solid-state lithium batteries using ultrathin polymer solid electrolytes.

## Supplementary Material



## Notes

SEM images, conductivities, and half-cell performance of PEO-LiTFSI and PEO-LiTFSI-PE electrolytes. Stress–strain curves of PEO-LiTFSI and PEO-LiTFSI-PE-Li. XPS results of O, N, and F elements of PEO-LiTFSI-PE. Critical current density test after formation of dead Li filler of PEO-LiTFSI-PE-Li. Interface resistance and electrochemical performance of Li||LiFePO_4_ full cells (PDF)
