# Supplementary material for: From Dead Lithium to Functional Fillers: An in Situ Conversion Strategy for High-Performance All-Solid-State Lithium Metal Batteries
Source: ACS Appl Mater Interfaces. 2026 Apr 7;18(15):21934–44. doi: 10.1021/acsami.5c25305 (PMC13107378; doi:10.1021/acsami.5c25305)
Supplement: Supplementary file 1 [file am5c25305_si_001.pdf]

Supporting Information

From dead lithium to functional fillers: an in-situ conversion strategy for high-performance all-solid-state lithium metal batteries

*Guoping Liu, Xinyu Zhang, and Maochun Wu**

Department of Mechanical Engineering, The Hong Kong Polytechnic University, Kowloon, Hong Kong SAR, 999077, China

* Corresponding author. E-mail: maochun.wu@polyu.edu.hk (M.C. Wu)

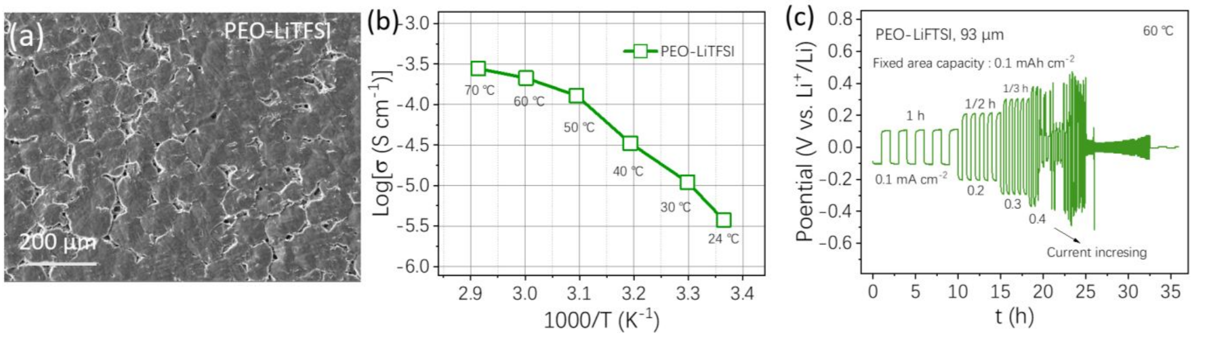

Figure S1. (a) SEM image of the PEO-LiTFSI solid electrolyte. (b) Arrhenius plots of Bare PEO-LiTFSI solid electrolyte. (c) Critical current density test of Li||PEO-LiTFSI||Li cell with the area capacity of 0.1 mA h cm^{-2}.

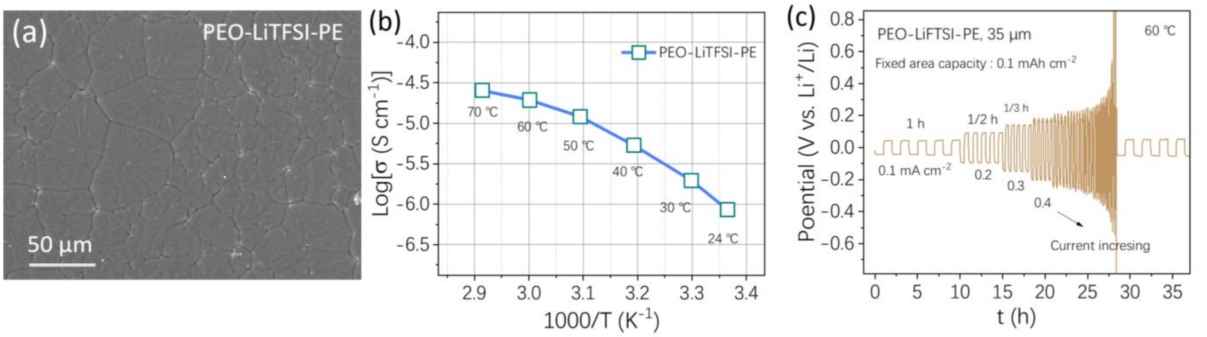

Figure S2. (a) SEM image of the PEO-LiTFSI-PE solid electrolyte. (b) Arrhenius plots of Bare PEO-LiTFSI-PE solid electrolyte. (c) Critical current density test of Li||PEO-LiTFSI-PE||Li cell with the area capacity of 0.1 mA h cm^{-2}.

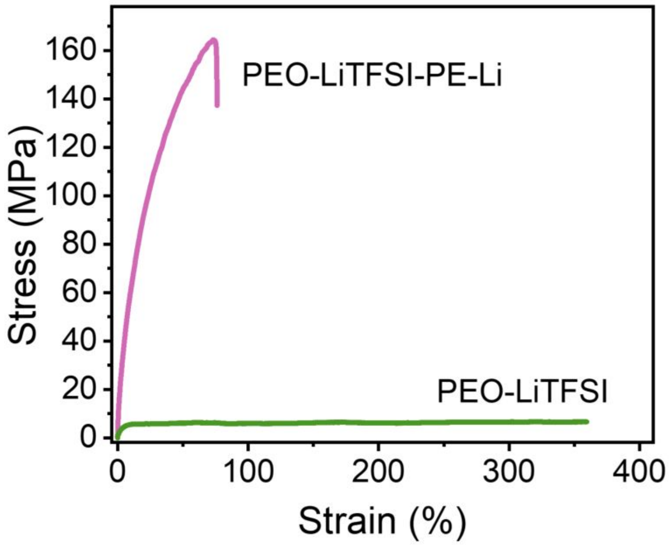

Figure S3. Stress−strain curves of PEO-LiTFSI and PEO-LiTFSI-PE-Li

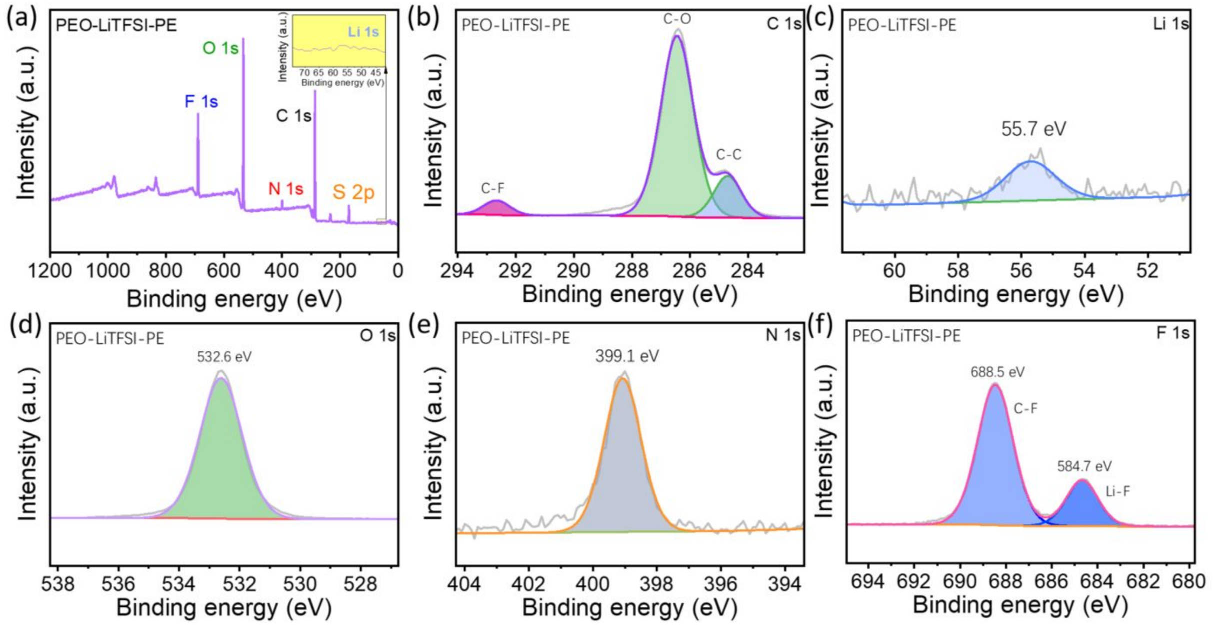

Figure S4. (a) Full XPS spectra of PEO-LiTFSI-PE. High-resolution XPS spectra of (b) C 1s, (c) Li 1s, (d) O 1s, (e) N 1s and (f) F 1s of PEO-LiTFSI-PE electrolyte.

Table S1. The content of C, N, O, F, and Li elements in PEO-LiTFSI-PE-Li, calculated from XPS results at different etching depths.

etching depth	C (atomic %)	F (atomic %)	Li (atomic %)	N (atomic %)	O (atomic %)
0 nm	44.84	10.24	20.22	1.11	19.58
10 nm	28.23	22.92	32.28	1.25	15.32
20 nm	26.00	24.44	33.07	1.24	15.26
30 nm	24.91	25.91	32.62	1.23	15.34
40 nm	23.53	26.33	33.95	1.20	14.98

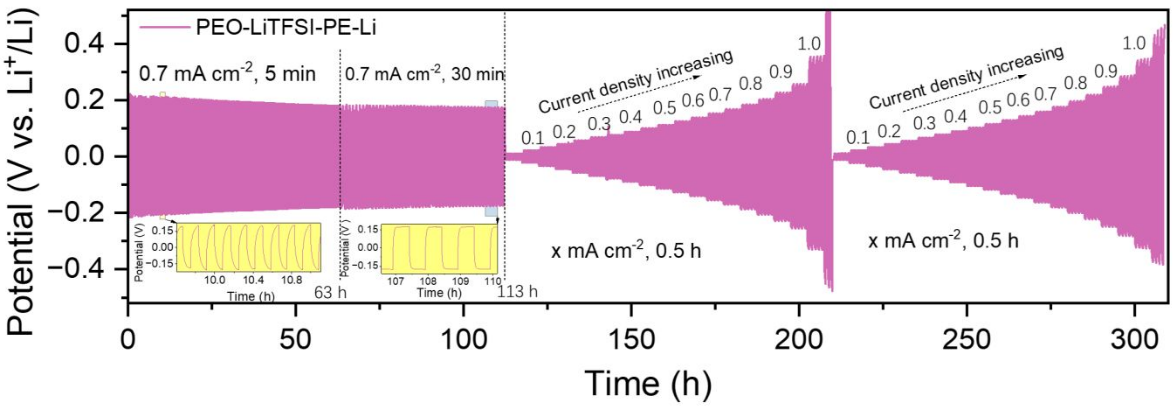

Figure S5**.** Critical current density test after formation of dead Li filler of PEO-LiTFSI-PE-Li.

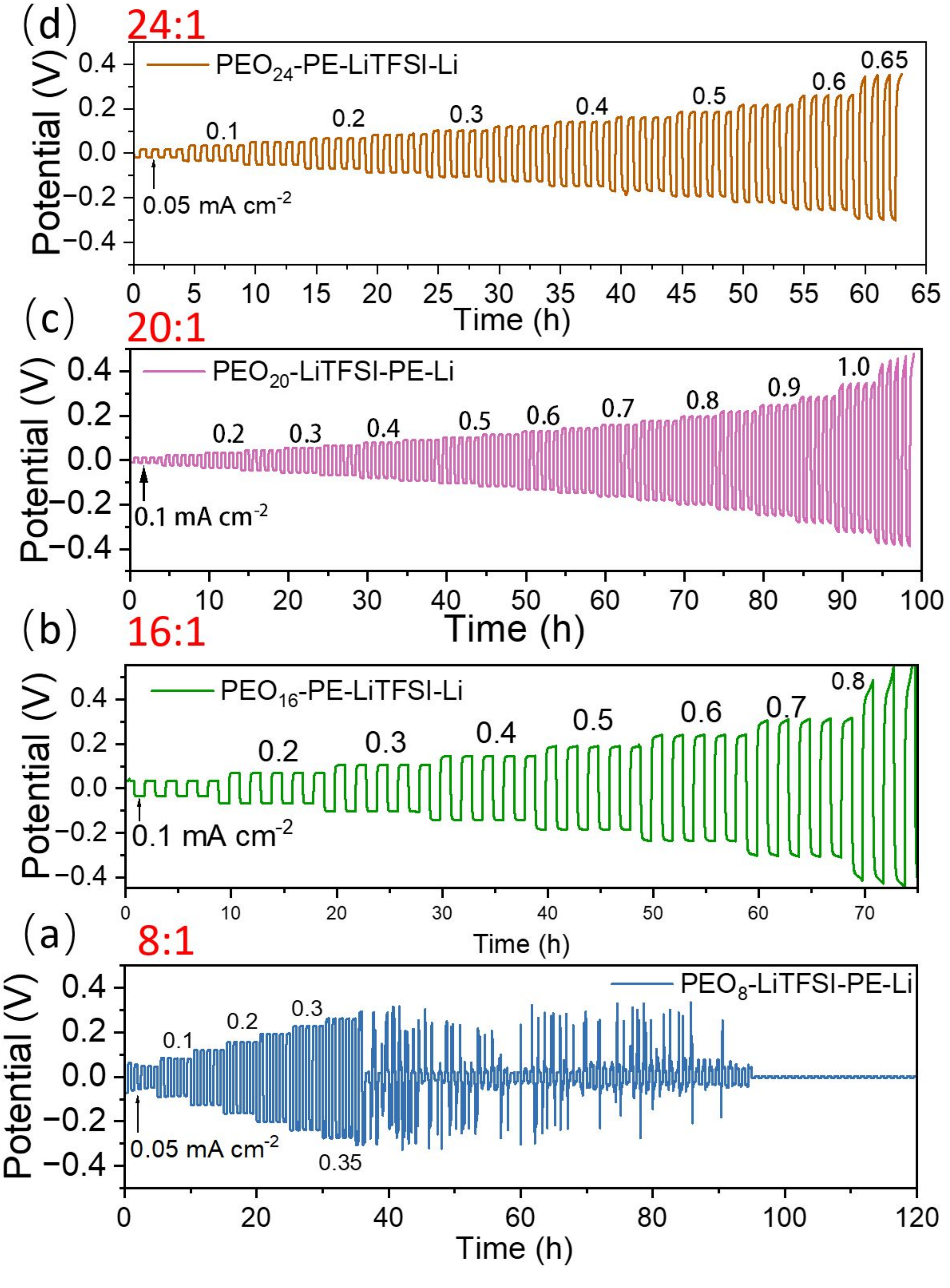

Figure S6. Critical current density tests of (a) PEO_8-LiTFSI-PE-Li, (b) PEO_{16}-LiTFSI-PE-Li, (c) PEO_{20}-LiTFSI-PE-Li and (d) PEO_{24}-LiTFSI-PE-Li.

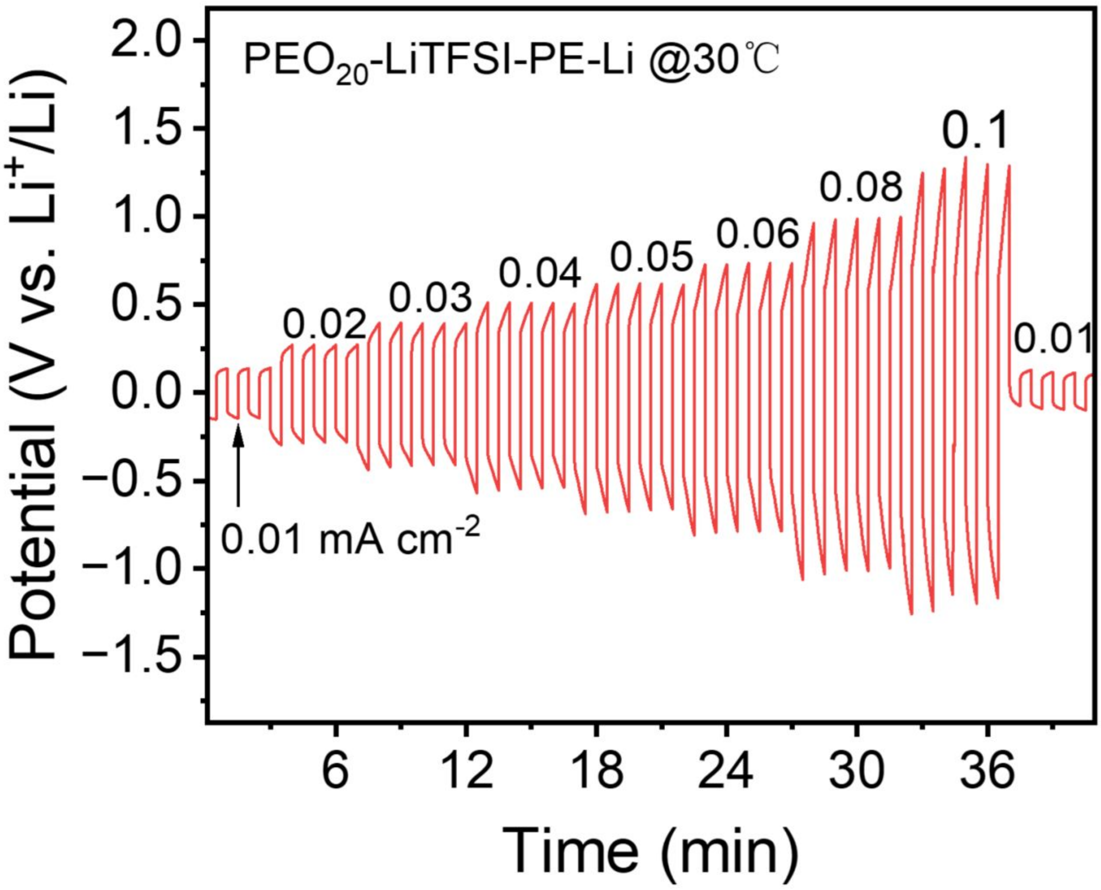

Figure S7. Critical current density test of PEO_{20}-LiTFSI-PE-Li at 30 ℃.

(a)

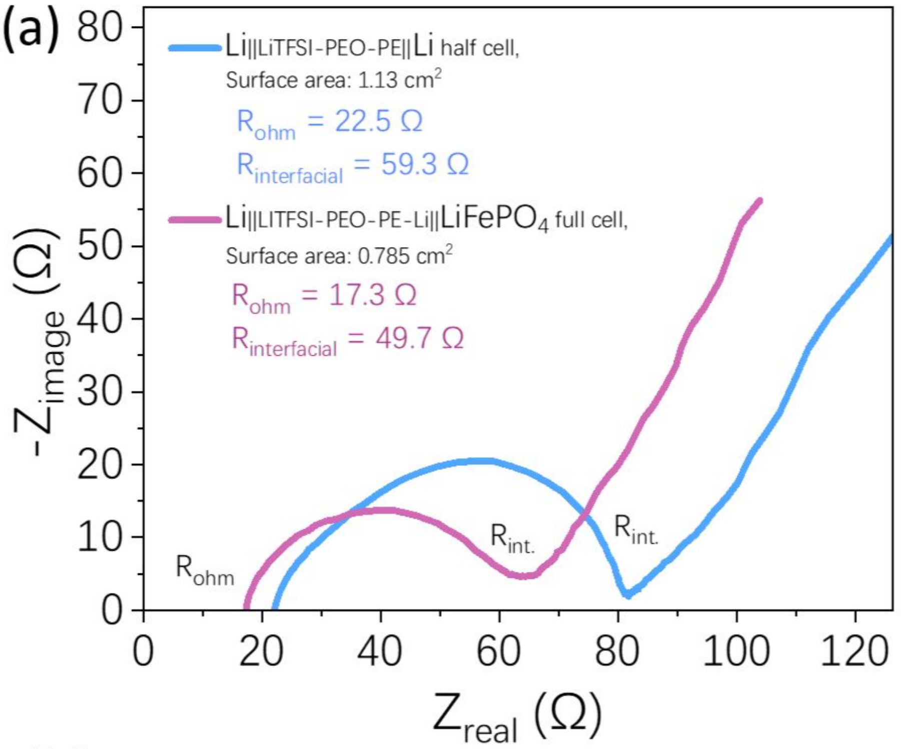

(b)

Interfacial resistance of the Li|LiFePO$_4$ full cell		
	Li|Li	Li|LiFePO$_4$
Overall R_{Int}	67.0 Ω cm^2	39.0 Ω cm^2
Anode R_{Int}	33.5 Ω cm^2	33.5 Ω cm^2
Cathode R_{Int}	-	5.5 Ω cm^2

Figure S8. (a) Nyquist plots of different Li||LiFePO$_4$ full batteries and (b) the calculated interface resistance.

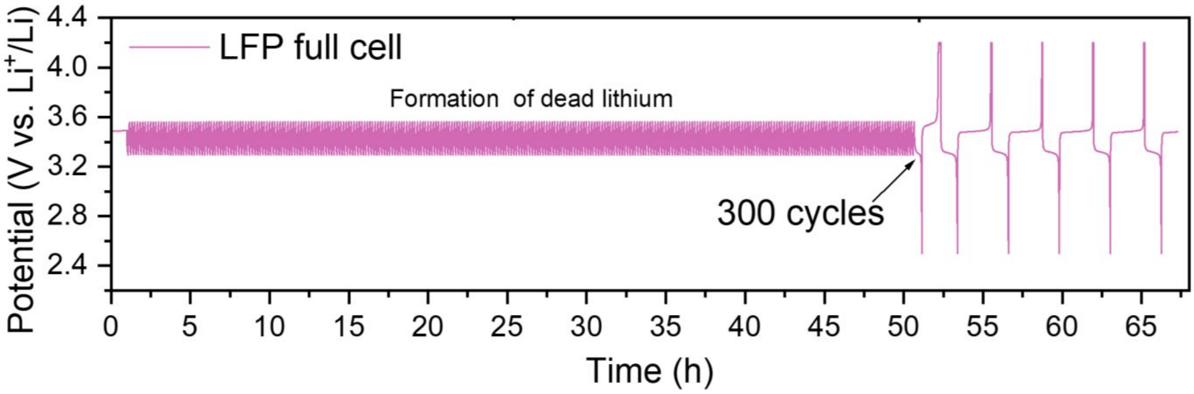

Figure S9. Formation of dead lithium in Li||$LiFePO_4$ before cycling.

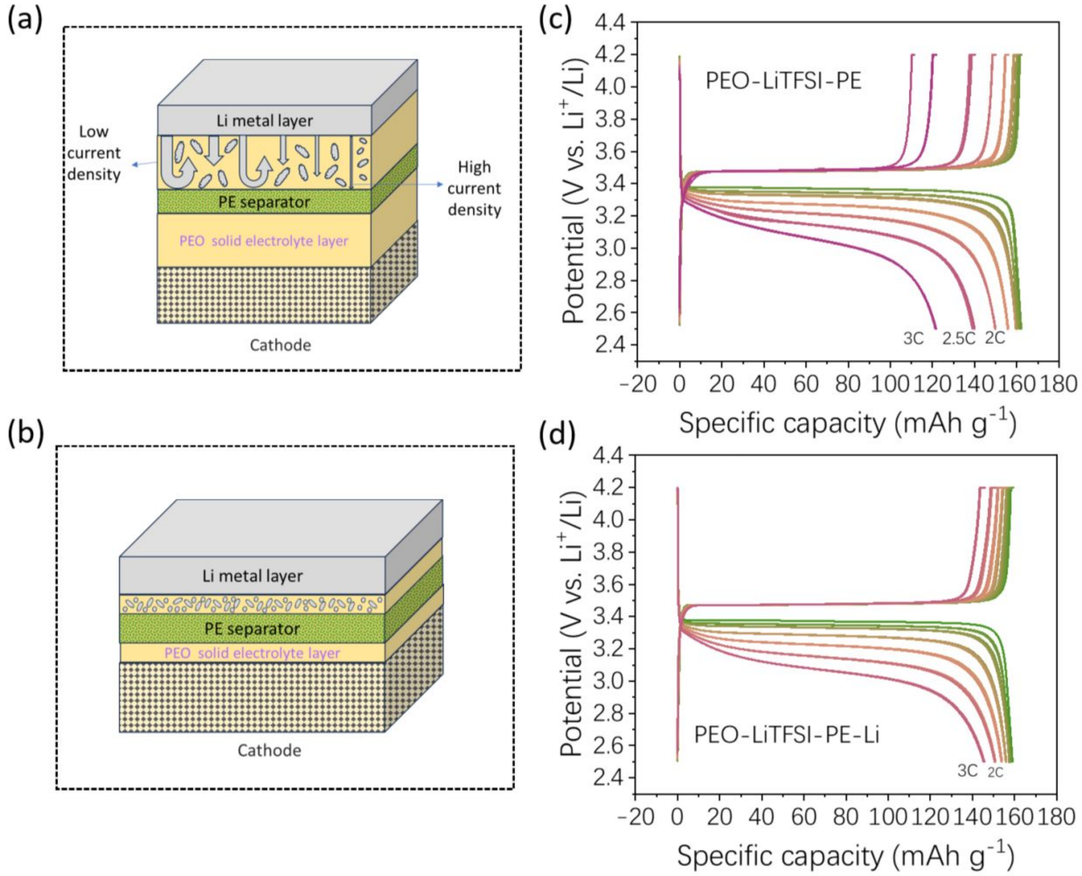

Figure S10. Structural diagram of (a) Li||PEO-LiTFSI-PE||$LiFePO_4$ and (b) Li||PEO-LiTFSI-PE-Li||$LiFePO_4$ full cell. Rate performance of (c) Li||PEO-LiTFSI-PE||$LiFePO_4$ and (d) Li||PEO-LiTFSI-PE-Li||$LiFePO_4$ full cells.

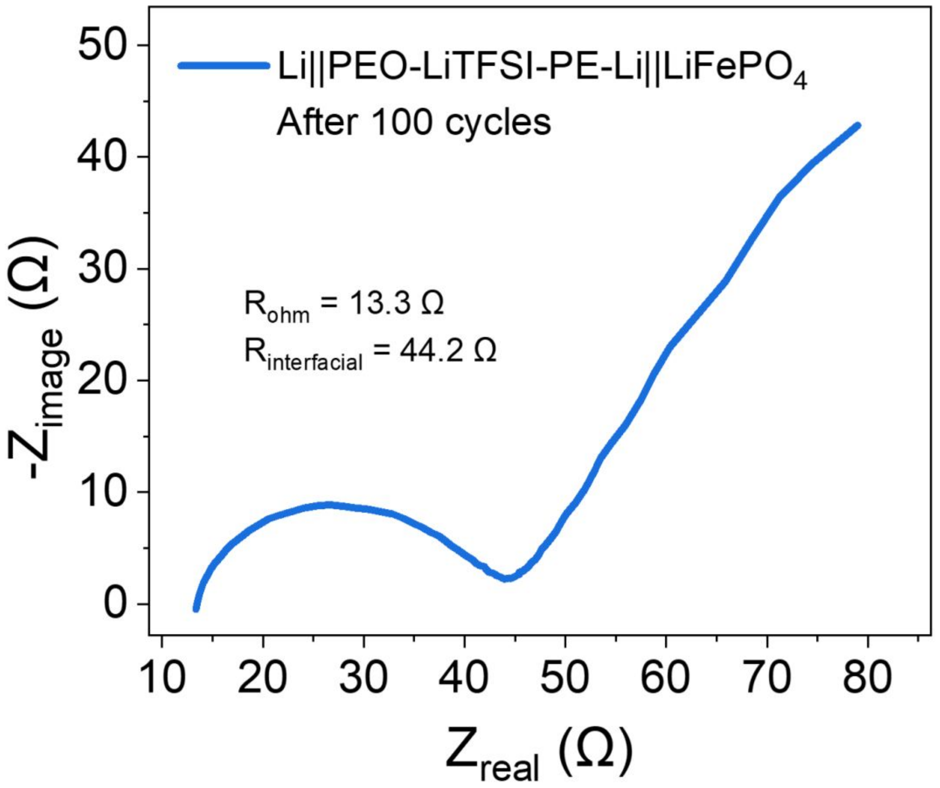

Figure S11. Electrochemical impedance spectroscopy (EIS) spectra of the Li||PEO-LiTFSI-PE-Li||$LiFePO_4$ cell after 100 cycles.

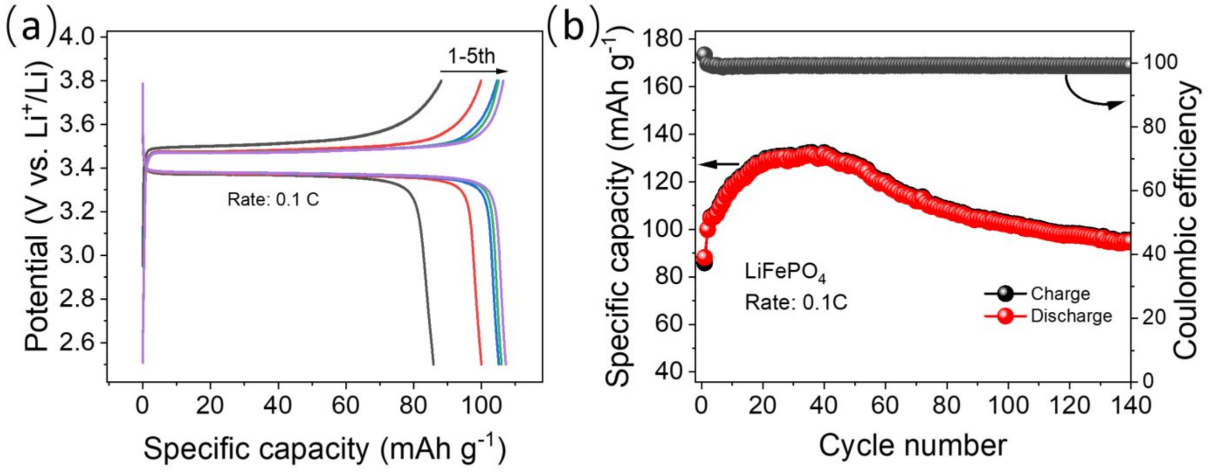

Figure S12. (a) The charge-discharge curves and (b) cycling stability of Li||PEO-LiTFSI-PE-Li||$LiFePO_4$ at room temperature (30 °C)

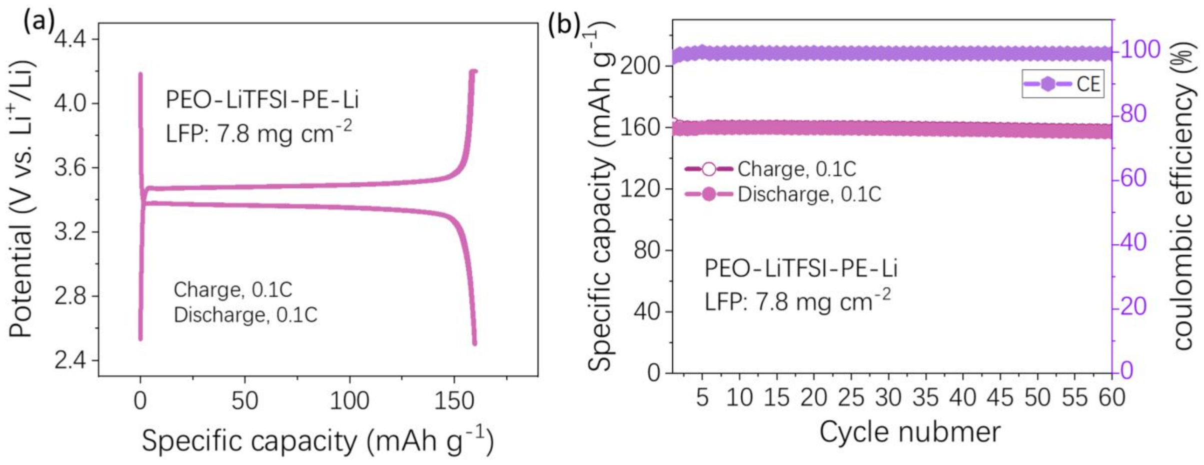

Figure S13. (a) Charge-discharge curve and (b) cycling performance of Li||PEO-LiTFSI-PE-Li||$LiFePO_4$ full cell with high mass loading.

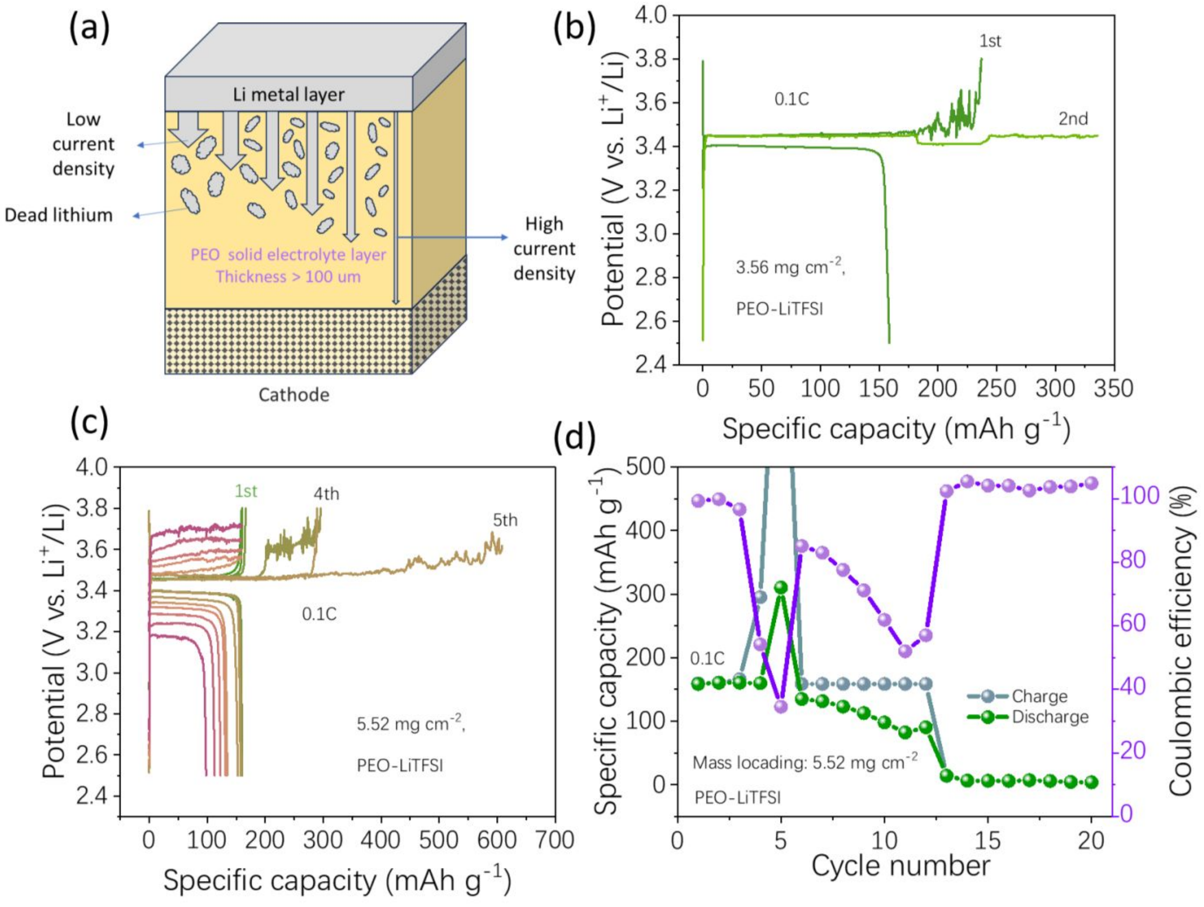

Figure S14. (a) Structural diagram of Li||PEO-LiTFSI||$LiFePO_4$ full cells. (b) charge-discharge curves of Li||PEO-LiTFSI||$LiFePO_4$ full cells. (c) Rate performance and (d) cycling performance of Li||PEO-LiTFSI||$LiFePO_4$ full cells.
